# Piezoelectric poly(3-hydroxybutyrate-co-3-hydroxyhexanoate) (PHBHHx) microspheres for collagen regeneration and skin rejuvenation

**DOI:** 10.3389/fbioe.2025.1554825

**Published:** 2025-03-12

**Authors:** Zeyu Fu, Yingwei Qu, Yinghao Wu, Yuan Xu, Hengdi Zhang, Yaozong Tang, Ziying Jin, Jia Zhao, Chang Tan

**Affiliations:** Beijing Joyinera Biomaterial Technology Co., Ltd., Beijing, China

**Keywords:** PHBHHx, LIPUS, piezoelectric, collagen regeneration, skin rejuvenation

## Abstract

**Introduction:**

Skin aging is an inevitable physiological process driven by factors like cellular senescence, ultraviolet radiation (UV) radiation, and environmental pollutants. A key feature is the accelerated collagen degradation in the dermal extracellular matrix, leading to visible signs such as sagging, wrinkles, and hyperpigmentation. Traditional fillers, such as hyaluronic acid and collagen-based fillers, offer only temporary volume enhancement without stimulating collagen regeneration. Studies have shown that electrical signals generated by piezoelectric materials can promote tissue regeneration.

**Methods:**

This study explored the potential of piezoelectric PHBHHx microspheres as an innovative skin filler for enhancing collagen regeneration and improving maxillofacial aesthetics, with the aid of low-intensity pulsed ultrasound (LIPUS) stimulation. A comprehensive characterizations of the piezoelectric PHBHHx microspheres were conducted, and their potential to stimulate collagen regeneration was assessed using a subcutaneous injection model in New Zealand white rabbits.

**Results:**

The results indicated that PHBHHx microspheres exhibited stable degradation properties, great piezoelectric properties and excellent biocompatibility. Moreover, when stimulated by LIPUS, the collagen-regenerating effect of PHBHHx microspheres was further enhanced, histological analysis revealed a denser and more organized collagen structures in LIPUS-stimulated PHBHHx group.

**Discussion:**

These findings highlight the potential of PHBHHx microspheres as an advanced biomaterial for applications in aesthetic medicine, particularly in promoting collagen regeneration and enhancing skin rejuvenation.

## 1 Introduction

Skin aging, characterized by a decline in functional capacity, is a gradual and unavoidable physiological process influenced by a combination of internal and external factors (Wang et al., 2024). These include cellular senescence, chronic UV exposure, and environmental pollutants (Shin et al., 2023). The accelerated degradation of collagen, a vital structural protein within the dermal extracellular matrix (ECM) (Zhang et al., 2023) essential for maintaining skin strength, firmness, and resilience (Huang et al., 2022), is widely acknowledged as a key contributor to visible signs of aging, including sagging skin, deep wrinkles, and hyperpigmentation (David and Jennifer, 2021). Therefore, promoting collagen regeneration has become a key objective in the development of new skin fillers that not only address the cosmetic appearance but also improve the underlying skin architecture (Pu et al., 2080). Traditional dermal fillers, such as hyaluronic acid (HA) (Fanian et al., 2023) and collagen-based fillers (Yang et al., 2024), primarily focus on restoring facial volume (Burgess et al., 2024), reducing wrinkles (Kim, 2021), and addressing other signs of aging. While these fillers can provide short-term improvements in skin appearance, they can’t offer long-term solutions, as their effects are transient and require repeated treatments (Narins et al., 2008). Furthermore, these fillers typically do not stimulate the body’s natural regenerative processes, particularly the production of collagen (Li et al., 2023; Kim and Sykes, 2011). This underscores the need for innovative biomaterials that not only offer volumizing benefits but also promote endogenous collagen regeneration.

Piezoelectric materials can generate electrical signals in response to mechanical stimulation such as pressure, vibration, or deformation (Badali et al., 2024). Studies have confirmed that these electrical signals can mimic natural cellular cues, thereby enhancing tissue healing and regeneration (Rajabi et al., 2015; Donate et al., 2023; Panda and Basu, 2021). Zhang et al. (2025) prepared nanocomposite electrospun dressings using poly (L-lactic acid) (PLLA) and barium titanate (BaTiO_3_) for scar-free wound recovery. The results demonstrated that ultrasound-induced activation of the piezoelectric effect reversed the fibrotic phenotype, resulting in scar-free healing and the regeneration of functional skin structures. Among the numerous piezoelectric materials, PHBHHx, a kind of intracellular polyhydroxyalkoxyfatty acid ester synthesized by many bacteria, has attracted wide attention due to its good biocompatibility, biodegradability and piezoelectricity (Bugnicourt et al., 2014). While PHBHHx has been extensively studied in various biomedical applications, including tissue engineering and drug delivery systems (Yang et al., 2022; Luo et al., 2024), its potential as a piezoelectric biomaterial for aesthetic applications remains largely unexplored. LIPUS is a type of pulsed ultrasound that uses a low intensity and output mode (Zheng et al., 2024). Due to its low intensity and pulsed output mode, LIPUS has minimal thermal effects while maintaining the transmission of acoustic energy to the target tissue. LIPUS has been shown to promote fresh, nonunion, or delayed union in animal models and clinical treatment (Donate et al., 2023). Studies have shown that LIPUS can effectively stimulate piezoelectric materials to generate electrical signals. Li et al. (2024) fabricated an ultrasound-responsive polyether ether ketone composite (PDA@BTO-SPEEK, PBSP) for repairing maxillofacial bone defects, utilizing the piezoelectric signal generated by barium titanate (BTO) under LIPUS stimulation, combined with the mediated effect of polydopamine (PDA). The results indicated that when PBSP is stimulated by LIPUS, it can generate stable electricity and effectively accelerate the osteogenic differentiation of osteoblasts.

**FIGURE 1:**
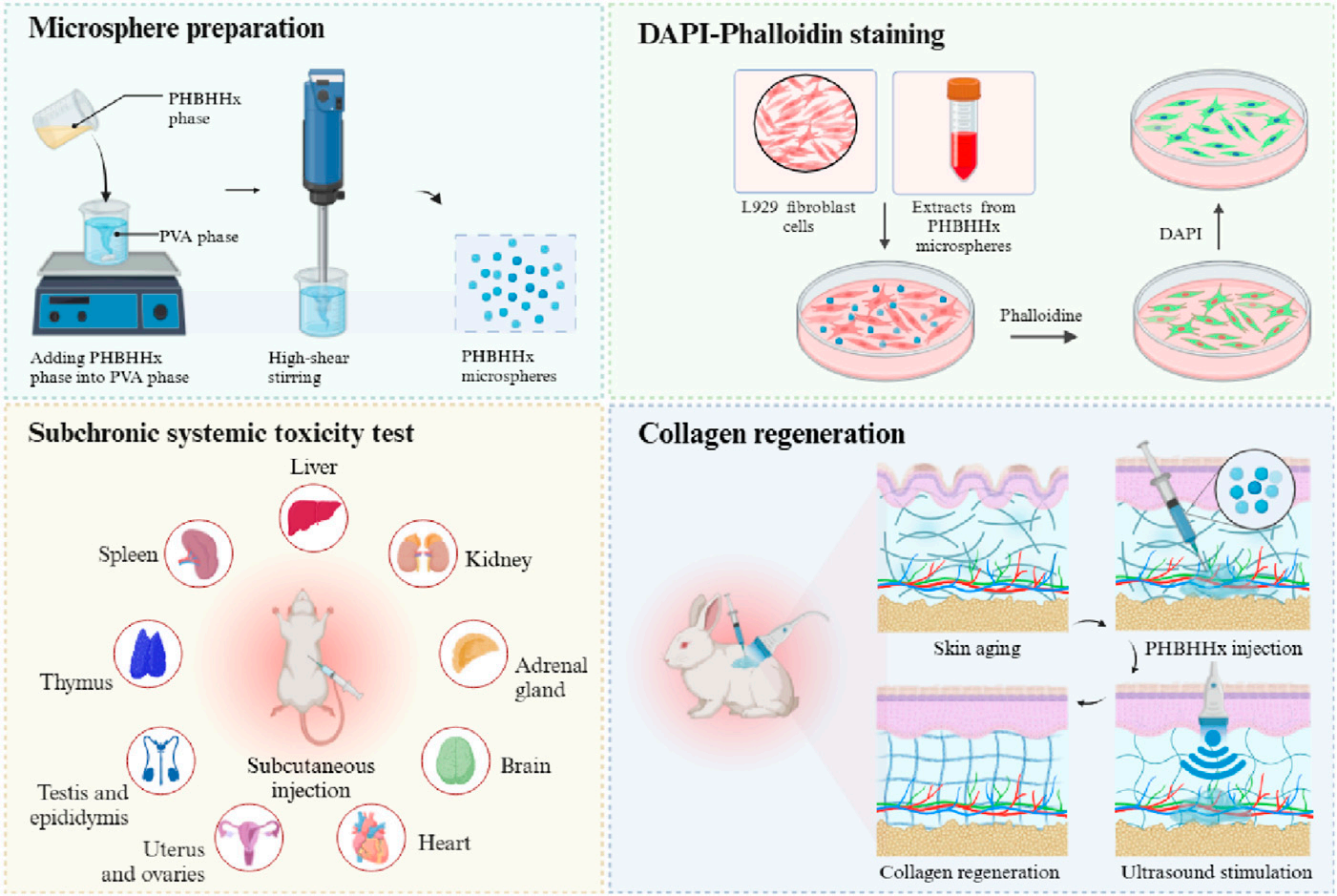
Schematic illustration of the preparation, *in vitro* and *in vivo* biocompatibility, and collagen regeneration properties of PHBHHx microspheres.

In this context, we hypothesized that the combination of PHBHHx with LIPUS could generate localized electrical signals to enhance skin collagen regeneration and achieve maxillofacial esthetics (Figure 1). To test this, we developed a subcutaneous injection model in New Zealand white rabbits to assess the effectiveness of LIPUS-stimulated PHBHHx microspheres on collagen production. The results indicated that PHBHHx microspheres had a diameter of 20–60 μm, exhibited stable degradation properties both *in vitro* and *in vivo*, demonstrated great piezoelectric properties and excellent biocompatibility. Moreover, the LIPUS-stimulated PHBHHx group exhibited significantly enhanced collagen production compared to other groups. Histological analysis revealed a denser and more organized collagen structures in LIPUS-stimulated PHBHHx group. These findings suggested that PHBHHx microspheres have significant potential as a skin filler in aesthetic medicine, while also advancing the development of bioactive fillers that promote tissue regeneration at the cellular level.

## 2 Materials and methods

### 2.1 Materials

The PHBHHx microspheres used in this study were prepared by Beijing Joyinera biomaterial Technology Co., Ltd. (Beijing, China). For *in vitro* biological tests, L929 fibroblasts were acquired from Chinese Academy of Sciences (Shanghai, China). Fluorescein isothiocyanate (FITC)-phalloidin and DAPI were provided by Beyotime (Shanghai, China). Minimum Essential Medium (MEM), Fetal Bovine Serum (FBS), Phosphate-buffered saline (PBS), 0.25% trypsin-EDTA and penicillin-streptomycin (PS) were provided by Gibco (United States). Hematoxylin and eosin were provided by SIGMA (Shanghai, China), Masson’s trichrome staining solution and Sirius Red staining Kit were provided by Solarbio (Beijing, China). Antibodies to Collagen I (Col-I) and Collagen III (Col-III) were provided by Bioss (Beijing, China). New Zealand white rabbits were supplied by Hangzhou Yuhang science union rabbit industry professional cooperatives (Hangzhou, China). SD rats were provide by Institute of Laboratory Animal Resources, Chinese Institute for Food and Drug Control (Beijing, China).

### 2.2 Preparation of PHBHHx microspheres and PHBHHx-CMC composite

The preparation of PHBHHx microspheres was conducted using an oil-in-water (O/W) emulsion solvent evaporation method (Tang et al., 2024). The process began with dissolving PHBHHx in dichloromethane to create the oil phase. This phase was then added dropwise to an aqueous solution of polyvinyl alcohol (PVA) under high-speed stirring, forming an emulsion. The emulsion was maintained under continuous stirring for several hours to allow the evaporation of the dichloromethane solvent, resulting in the formation of solid microspheres. The microspheres were then collected by filtration, followed by multiple washes with distilled water to remove any residual PVA. In addition, a vibrating sieving technique was employed to obtain microspheres with diameters ranging from 20 to 60 μm. In addition, to facilitate the subcutaneous injection of PHBHHx microspheres, a PHBHHx-carboxymethyl cellulose (CMC) composite was prepared by thoroughly stirring PHBHHx microspheres with CMC solution at a ratio of 300 mg of PHBHHx microspheres per 1 mL of CMC solution.

### 2.3 Preparation of porous and solid PHBHHx membranes

Porous and solid PHBHHx membranes were fabricated using electrospun technology by modulating the solution flow rate and electrode voltage. Prior to electrospun, 3 g PHBHHx was added into 10 mL of hexafluoroisopropanol and stirred overnight to obtain a homogeneous solution. For the preparation of porous membranes, a solution flow rate of 0.5 mL/h was utilized, along with a negative electrode voltage of 8Kv. In contrast, solid membranes were produced by increasing the flow rate to 2.5 mL/h while applying a negative electrode voltage of 14Kv, and maintaining a consistent positive electrode voltage of 12Kv for both membrane types. The collection drum was rotated at a speed of 30 r/min throughout the electrospun process.

### 2.4 Characterization of PHBHHx microspheres and PHBHHx-CMC composite

Scanning electron microscopy (SEM, Zeiss) was employed to examine the surface morphology of PHBHHx microspheres, as well as the structural characteristics of PHBHHx-CMC composite. For the PHBHHx-CMC composite, an oven drying treatment was required to remove water prior to detection. Samples were coated with a thin layer of gold before imaging. In addition, the particle size distribution of PHBHHx microspheres was measured using a laser diffraction particle size analyzer (Malvern Mastersizer 3000, UK). Fourier Transform Infrared Spectroscopy (FTIR, Thermo) was used to confirm the chemical structure of both the PHBHHx microspheres and the PHBHHx-CMC composite. The FTIR spectra were recorded in the range of 400–4000 cm^−1^.

The piezoelectric properties of PHBHHx microspheres were characterized using piezoresponse force microscopy (PFM, Bruker Dimension Icon, Germany). Samples were prepared by dispersing PHBHHx microspheres in ethanol and then dropping onto silicon wafer. After drying, PFM measurements were conducted in contact mode, where an polarization voltage (-10-10V) was applied to a conductive AFM tip in contact with the sample surface.

To evaluate the mechanical properties and delivery efficacy of the PHBHHx-CMC composite as a potential filler for subcutaneous injection in aesthetic applications, the composite was loaded into a 1 mL syringe and injected with an 18-gauge needle (inner diameter: 1.2 mm) and secured in a specialized mold. A universal material testing machine was then employed to apply vertical load compression and pushing at a constant speed of 30 mm/min. The pushing force-displacement curve was recorded, with each sample tested in triplicate to ensure the reliability of the results.

The rheological property provided insight into the injectability of materials, which is critical for their application in skin filling. The rheological behavior of the PHBHHx-CMC composite was evaluated using a rotational rheometer (Haake Mars60, Germany). Measurements were performed at varying shear rates to determine the viscosity and flow properties of the composite. Briefly, a volume of 0.2 mL of the solution was carefully applied to the test plate using a 1 mL syringe, ensuring a consistent test height of 1 mm. Following the positioning of the PP25 rotor at the designated test site, any excess solution was aspirated, and the test was initiated. The experiments were conducted at a controlled temperature of 25°C, with shear rates varying from 0.1 S^−1^ to 100 S^−1^. The viscosity curve of the solution was recorded, with each sample evaluated in duplicate across three independent trials to ensure consistent reproducibility.

To determine the stability and longevity of PHBHHx as a biomaterial for potential applications in tissue engineering and aesthetic medicine, degradation studies were conducted both *in vitro* and *in vivo*. *In vitro* degradation was assessed by immersing the PHBHHx microspheres in phosphate-buffered saline (PBS) at temperature of 37°C and 60°C, with measurements of molecular weight (Mw) loss and morphological changes recorded at predetermined time points. For *in vivo* degradation, porous and solid PHBHHx electrospun membranes were subcutaneously implanted in the back of New Zealand white rabbits. After death was induced by intraperitoneal injection of Sutai ^®^50 (50 mg/kg body weight) at predetermined time points, the solid and porous membranes were removed and analyzed for morphological structure and molecular weight loss.

### 2.5 Biocompatibility of PHBHHx microspheres and PHBHHx-CMC composite

To assess the biocompatibility of PHBHHx microspheres, fibroblasts were cultured with extracts from microspheres for 24 and 48 h. After the incubation period, the cells were stained with DAPI and FITC-phalloidin to evaluate cell morphology and cytoskeletal structure. Additionally, a subchronic systemic toxicity test was conducted to further verify the biocompatibility of PHBHHx-CMC composite. After 1 week of breeding in the animal laboratory, rats were randomly divided into two groups based on body weight, with 20 SD rats in each group, half male and half female. In the experimental group, the PHBHHx-CMC composite was implanted subcutaneously on the back of the rats at a dosage of 0.4 mL/100 g body weight, with 4 injection sites per SD rat (sample group). In the control group, SD rats received 0.9% sodium chloride solution through the same injection method (control group). The injections were administered once a month for a total of 3 times, with a 90-day exposure period. Prior to dissection, SD rats were fasted for 16 h, and their body weight was measured after fasting. Anesthesia was induced by intraperitoneal injection of Sutai^®^50 at a dose of 50 mg/kg body weight, followed by blood collection from the abdominal aorta to measure hematological and biochemical parameters. After blood collection, euthanasia was performed under anesthesia, and organ abnormalities were observed. The wet weights of various organs were measured, and organ coefficients (g/100 g) were calculated. The tissues were then fixed in 4% formaldehyde solution for routine histopathological examination.

### 2.6 PHBHHx-CMC composite used for collagen regeneration

In this study, New Zealand white rabbits were used to assess the efficacy of PHBHHx-CMC composite as a skin filler. The rabbits were randomly assigned to four experimental groups: (1) group receiving 0.9% sodium chloride solution injection (Blank), (2) group receiving PHBHHx-CMC injection (PHBHHx), (3) group receiving PHBHHx-CMC composite injection and LIPUS stimulation (PHBHHx + US) and (4) group receiving 0.9% sodium chloride solution injection and LIPUS stimulation (US). After anesthetization, two injection sites were marked along the dorsal surface of each rabbit (n = 2), ensuring uniform placement and distance between injection points. In the LIPUS-stimulated groups, LIPUS was applied at a frequency of 1 MHz and an intensity of 0.3W/cm^2^ for 20 min, 5 days per week, 1 week after injection. Tissue samples were harvested at 1, 2, 3, 5, and 13 weeks post-injection. Collected tissue samples were fixed in 4% formaldehyde solution, embedded in paraffin, and prepared into 4 μm sections for histopathological evaluation. Hematoxylin and eosin (H&E) staining was used to assess inflammatory response and overall tissue morphology. Masson’s trichrome staining was performed to visualize collagen deposition and distribution, while Sirius red staining, under polarized light, was used to differentiate between type I and type III collagen fibers. In addition, the expression of type I and type III collagen was characterized by immunohistochemical staining. All stained sections were analyzed and quantitative measurements of collagen deposition and inflammatory markers were performed using ImageJ software.

### 2.7 Statistic analysis

The data was shown as mean ± standard deviation (SD). Data were analyzed by one-way analysis of variance (ANOVA). Differences at P < 0.05 were considered statistically significant. The data were statistically analyzed by GraphPad Prism 5.0.

## 3 Results and discussions

### 3.1 Characterization of PHBHHx microspheres and PHBHHx-CMC composite

**FIGURE 2:**
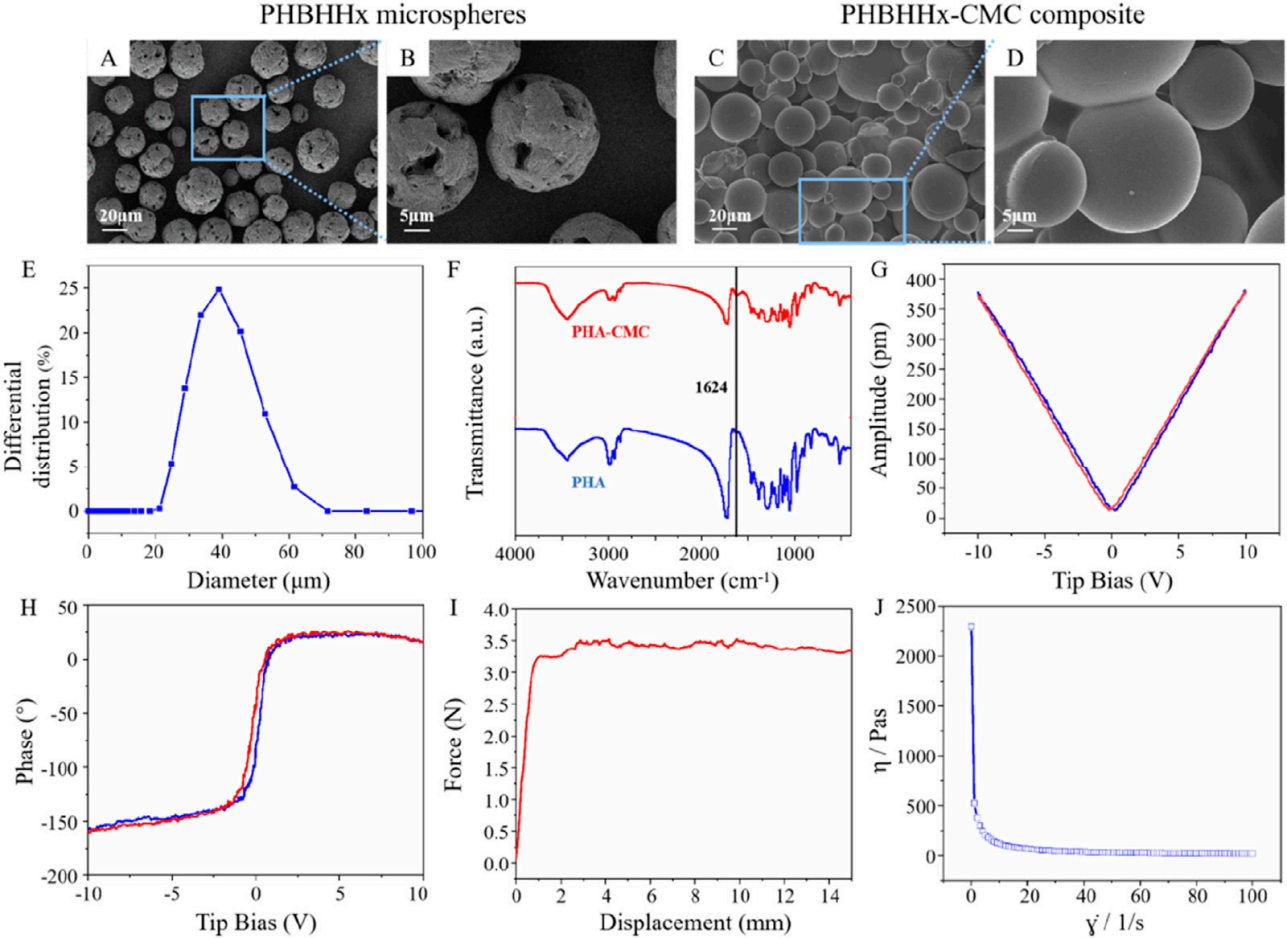
Characterization of PHBHHx microspheres and PHBHHx-CMC composite. SEM images of PHBHHx microspheres **(A, B)** and PHBHHx-CMC composite **(C, D)**. **(E)** Particle size distribution of PHBHHx microspheres. **(F)** FTIR spectrum of PHBHHx and PHBHHx-CMC composite. Piezoelectric properties of PHBHHx microspheres **(G, H)**. **(I)** The pushing force of PHBHHx-CMC composite. **(J)** The viscosity curve of PHBHHx-CMC composite.

SEM was used to characterize the microstructure of PHBHHx microspheres and PHBHHx-CMC composite. Images revealed that the PHBHHx microspheres exhibited a rough surface with microporous structure and uniform spherical shape (Figures 2A, B). This surface structure enhances the available surface area for interaction with surrounding tissues, which is beneficial for promoting cell adhesion (Ranella et al., 2010). The diameter distribution of PHBHHx microspheres ranged from 20 to 60μm, with an average diameter of 35.365 μm (Figure 2E). The PHBHHx-CMC composite, on the other hand, displayed smoother surfaces due to the encapsulating effect of the CMC layer, which provide additional mechanical support and improved injectability of the composite (Figure 2C). Additionally, membrane-like junctions formed by CMC can be observed between the PHBHHx microspheres (Figure 2D).

FTIR analysis confirmed the successful mixing of CMC and PHBHHx in PHBHHx-CMC composite, as evidenced by the appearance of characteristic absorption bands corresponding to CMC and PHBHHx (Figure 2F). In PHBHHx, the peak at 3445 cm^−1^ corresponds to O-H stretching vibration, while the peaks at 2,965 cm^−1^ and 2,935 cm^−1^ are attributed to C-H stretching vibrations. The peak at 1726 cm^−1^ indicates C=O stretching vibration, and the peaks at 1,464 cm^−1^ and 1,385 cm^−1^ are associated with C-H bending vibrations. The peak at 1,286 cm^-1^ corresponds to C-O stretching vibration, and the peaks at 1,185 cm^−1^, 1,135 cm^−1^, 1,096 cm^−1^, and 1,055 cm^−1^ are attributed to C-O-C stretching vibrations. In PHBHHx-CMC composite, in addition to the absorption peaks characteristic of PHBHHx, a peak at 1,624 cm^−1^ corresponding to the C=O stretching vibration in CMC was also observed, confirming the successful integration of PHBHHx and CMC.

The piezoelectric properties of PHBHHx microspheres were evaluated using PFM. The piezoelectric response of PHBHHx microspheres was evaluated by phase-tip shift and amplitude-tip shift maps. The amplitude-tip bias mapping showed a linear increase in piezoresponse with increasing bias voltage, confirming the electromechanical coupling effect (Figure 2G). The phase-tip bias mapping revealed clear polarization behavior, indicating the presence of piezoelectric domains within the PHBHHx microspheres (Figure 2H). These results suggested that the PHBHHx microspheres exhibited stable piezoelectric characteristics.

The extrusion force of dermal fillers is a critical parameter that directly influences the ease of injection and patient comfort during aesthetic procedures (Pierre et al., 2015). It is determined by factors such as the viscosity (Bakrani Balani et al., 2023), cohesiveness (Alghamdi et al., 2019), and particle size of the filler material, as well as the gauge of the needle or cannula used. The extrusion force of PHBHHx-CMC composite was measured using an 18G needle. Initially, a rapid increase in force was observed as displacement began. As displacement continued to increase, the curve reaches a plateau at approximately 3.5N, indicating that a steady-state flow was achieved. The stabilization of force suggested that the filler material possesses consistent rheological properties once initial resistance was overcome. This behavior is critical for practical applications, as it implies that the filler can be injected with predictable and controlled force, minimizing patient discomfort and ensuring precise material delivery during aesthetic procedures (Figure 2I). The viscosity test results of PHBHHX-CMC indicated that the viscosity decreased sharply with the increase of shear rate, highlighting the non-Newtonian shear thinning property of the composite. Viscosity remained high at low shear rates but decreased significantly as shear rates approached 100 S^−1^. These results indicated that the viscosity of the material can be controlled by the shear rate, which is more conducive to achieving controlled injection in clinical applications (Figure 2J).

**FIGURE 3:**
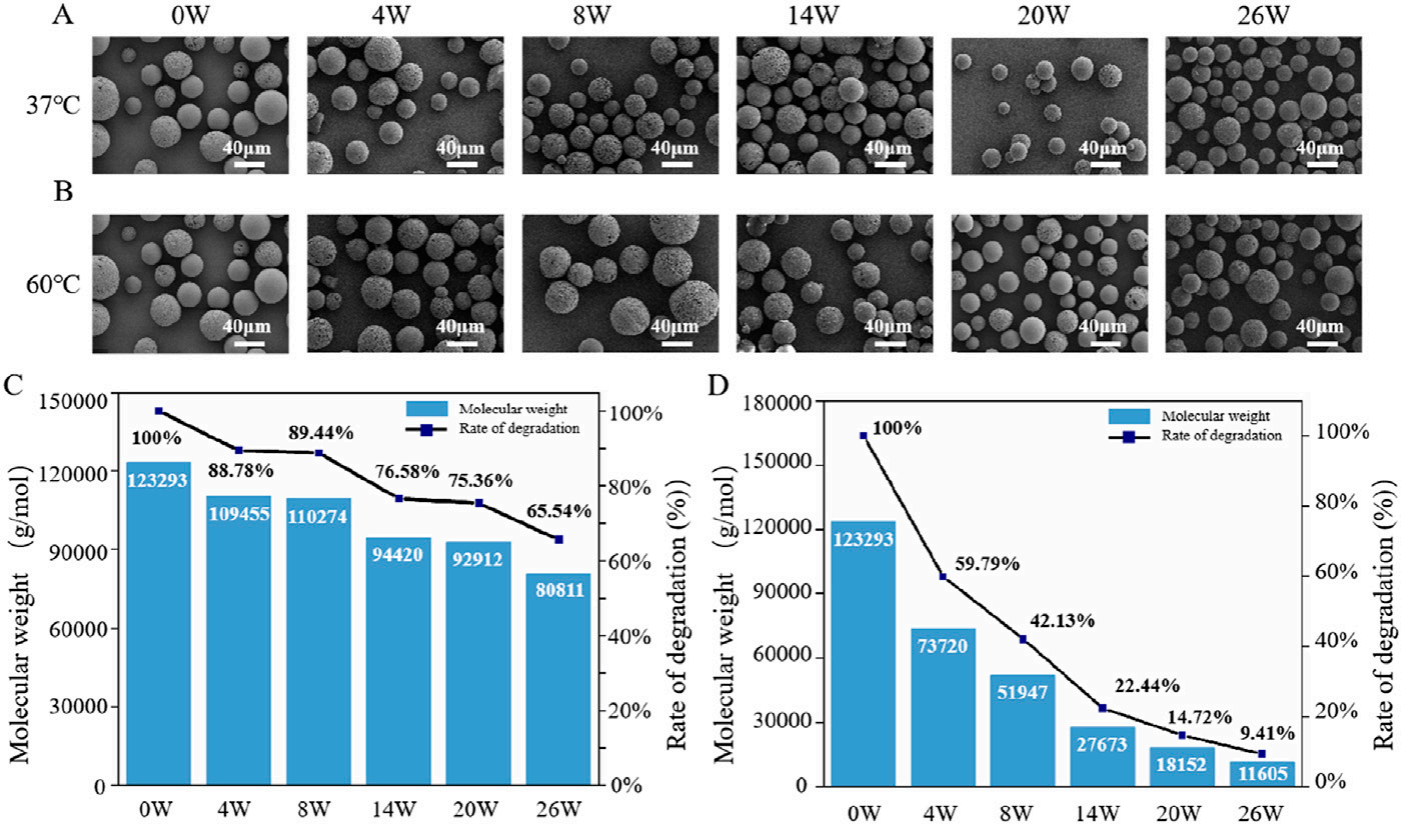
The *in vitro* degradation properties of PHBHHx microspheres. The morphological changes of PHBHHx microspheres in PBS at 37°C **(A)** and 60°C **(B)** The Mw changes of PHBHHx microspheres in PBS at 37°C **(C)** and 60°C **(D)**.

The degradation behaviors of PHBHHx microspheres in PBS at 37°C and 60°C were systematically evaluated. The results indicated that PHBHHx microspheres retained their spherical structure without signs of cracking or collapse for up to 26 weeks at both 37°C and 60°C (Figures 3A, B), demonstrating high structural stability under these conditions. Unlike synthetic polymers such as polylactic acid (PLA) and polycaprolactone (PCL), PHAs primarily degrade through surface erosion (Pouton and Akhtar, 1996), this characteristic allows PHA materials to retain their structural integrity for a longer duration. Molecular weight analysis indicated that, at 37°C, the Mw of PHBHHx microspheres decreased to 65.54% of the initial value, dropping from 123,293 to 80,811 by week 26 (Figure 3C). In contrast, at 60°C, the Mw decreased more dramatically, falling to 9.41% of the initial value, from 123,293 to 11,605 (Figure 3D). Theses results highlight the temperature-dependent acceleration of degradation.

### 3.2 Biocompatibility of PHBHHx microspheres and PHBHHx-CMC composite

**FIGURE 4:**
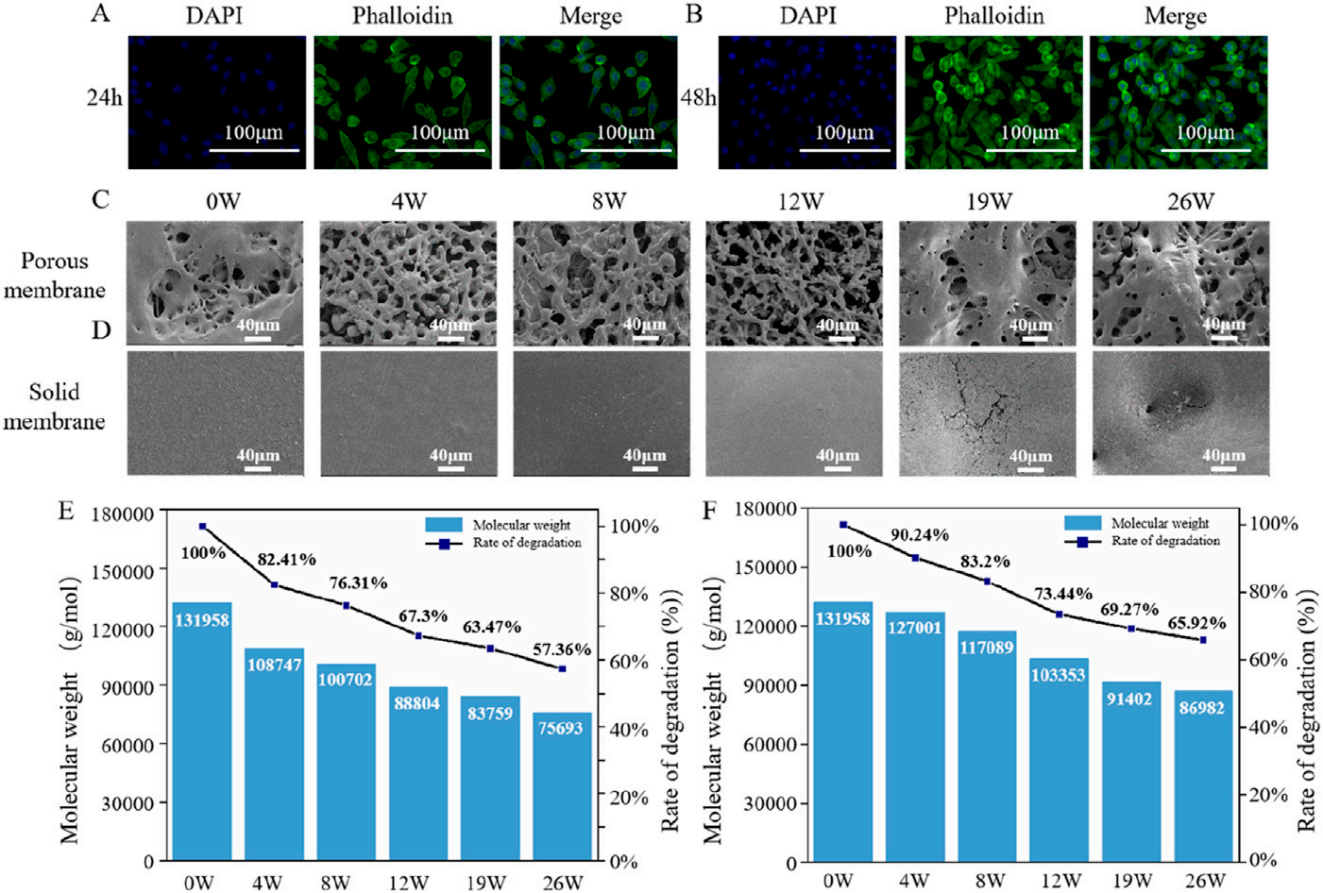
*In vitro* cytocompatibility and *in vivo* degradation properties of PHBHHx microspheres. DAPI-phalloidin staining of L929 cells *co*-cultured with extracts from PHBHHx microspheres for 24 h **(A)** and 48 h **(B)** The morphological changes of porous **(C)** and solid PHBHHx membranes **(D)** The Mw changes of porous **(E)** and solid **(F)** PHBHHx membranes.

Biomaterials play a crucial role in biomedical applications, and their biological characteristics are essential in determining their suitability for clinical use. Among these, biocompatibility is a key property that directly influences the material’s interaction with surrounding tissues and cells (Xu H. et al., 2024). Evaluating biocompatibility not only helps ensure minimal adverse reactions but also assesses the material’s ability to support cellular activities such as adhesion, proliferation, and differentiation. To investigate this, an *in vitro* cytocompatibility assay of PHBHHx microspheres was conducted. The results showed that after 24 and 48 h of co-culture with extracts from PHBHHx microspheres, L929 fibroblasts displayed distinct nuclear and cytoskeletal staining, as evidenced by DAPI and FITC-phalloidin labeling, respectively. DAPI staining revealed intact, well-defined nuclei with no signs of significant nuclear condensation or fragmentation, indicating maintained cellular viability. Phalloidin staining demonstrated a well-organized actin cytoskeleton with distinct filamentous structures, suggesting normal cytoskeletal integrity. Additionally, a notable increase in cell proliferation was observed at 48 h compared to 24 h (Figures 4A, B). No significant differences were observed in the nuclear and cytoskeletal morphology between 24 and 48 h, highlighting the excellent biocompatibility of the PHBHHx microspheres. PHBHHx degrades via surface erosion, resulting in minimal immune response (Lizarraga Valderrama et al., 2018). Furthermore, its degradation product, 3HB, is a natural component of blood (Miyazaki et al., 2015), which further explains the excellent biocompatibility of PHBHHx.

To further assess the biocompatibility of the PHBHHx-CMC composite, its subcutaneous systemic toxicity was evaluated through a 90-day implantation study in SD rats. The results showed that during the experiment, the rats in the control group and the sample group had normal appearance and behavior, and moved freely. The weight gain of the sample and control groups was normal during the test period, and there was no significant difference between the two groups of the same sex (Supplementary Table S1; Supplementary Figure S1). There was no significant difference in the average 24-h feed consumption per 100 g body weight per week between the two groups of the same sex (Supplementary Table S2; Supplementary Figure S2). The absolute value of lymphocytes (LYMPH10^9^/L) and the percentage of eosinophils (EO%) of male rats in the sample group were significantly different from those in the control group (P < 0.01). The percentages of neutrophil polymorphonuclear cell (NEUT%) and lymphocyte (LYMPH%) were significantly different between the two groups (P < 0.05). The activated partial thromboplastin time (APTT Sec) of female rats in the sample group was significantly different from that in the control group (P < 0.05), and there was no significant difference in the other indicators between the two groups of the same gender (Supplementary Table S3; Supplementary Figure S3). Alanine aminotransferase (ALT) of female rats in the sample group was significantly different from that in the control group (P < 0.01). Aspartate aminotransferase (AST) in female rats was significantly different from that in the control group (P < 0.05). There was no significant difference in other indicators between the two groups of the same gender (Supplementary Table S4; Supplementary Figure S4). There was no significant difference in the wet weight of organs between the sample group of the same sex and the control group (P > 0.05) (Supplementary Table S5). There was no significant difference in organ coefficient between the sample group and the control group of the same gender (P > 0.05) (Supplementary Table S6). Hematological analysis revealed significant differences in male rats, with the absolute lymphocyte count (LYMPH10^9^/L) and eosinophil percentage (EO%) being highly significant (P < 0.01), and neutrophil percentage (NEUT%) and lymphocyte percentage (LYMPH%) showing significant differences (P < 0.05) compared to controls. In female rats, activated partial thromboplastin time (APTT Sec) also differed significantly (P < 0.05). However, all values remained within the laboratory reference range, suggesting no biological significance. Biochemical analysis in female rats showed significantly higher alanine aminotransferase (ALT) (P < 0.01) and aspartate aminotransferase (AST) (P < 0.05) levels, yet these too were within the reference range, indicating no toxic effects. Histopathological examination revealed no tissue or organ damage associated with the composite. Overall, no signs of systemic toxicity, mortality, or adverse reactions were observed, and statistical analysis of all parameters, including organ weights and histopathology, indicated no clinically meaningful abnormalities or target organ toxicity related to the PHBHHx-CMC composite under the conditions of this study (Supplementary Table S7). No obvious toxic reaction or any signs of dying rats caused by PHBHHx-CMC composite were observed. After statistical analysis and comprehensive analysis of the results of hematology, blood biochemical indexes, organ wet weight, organ coefficient and histopathology, it was concluded that PHBHHx-CMC composite did not show clinically significant abnormal indicators or toxic target organs, indicating good biocompatibility.

In conclusion, both PHBHHx microspheres and the PHBHHx-CMC composite exhibited remarkable biocompatibility, as demonstrated by a comprehensive evaluation of their effects *in vitro* and *in vivo*. *In vitro* studies revealed no significant cytotoxicity, with fibroblasts maintaining normal morphology and viability when cultured with extracts from these materials. Similarly, *in vivo* assessments, including long-term implantation studies in SD rats, confirmed the absence of systemic toxicity or adverse local tissue reactions. These findings underscore the potential of PHBHHx microspheres as safe and effective biomaterials for biomedical applications.

### 3.3 *In vivo* degradation properties of PHBHHx porous and solid membranes

After demonstrating the biosafety of PHBHHx, the degradation properties of PHBHHX *in vivo* were investigated. The results indicated that electrospun porous PHBHHx membranes exhibited morphological changes as early as the fourth week, with an increasing number of broken fibers over time, characterized by smooth fracture surfaces (Figure 4C). Solid PHBHHx membranes underwent progressive surface smoothing, with surface cracks becoming evident by week 19 (Figure 4D). The primary reason for PHBHHx degradation *in vivo* is the presence of various enzymes in the body that can break its molecular chains, leading to gradual decomposition. *In vivo*, electrospun porous membranes demonstrated a faster reduction in molecular weight compared to solid membranes, by 26W, the Mw of electrospun porous membranes decreased to 57.36% of its initial value, from 131,958 to 75,693 (Figure 4E), while solid films decreased to 65.92% of its initial value, from 131,958 to 86,982 (Figure 4F), this is due to the fact that the porous structure increases the contact area with the environment, thereby accelerating degradation.

### 3.4 PHBHHx-CMC composite used for collagen regeneration

**FIGURE 5:**
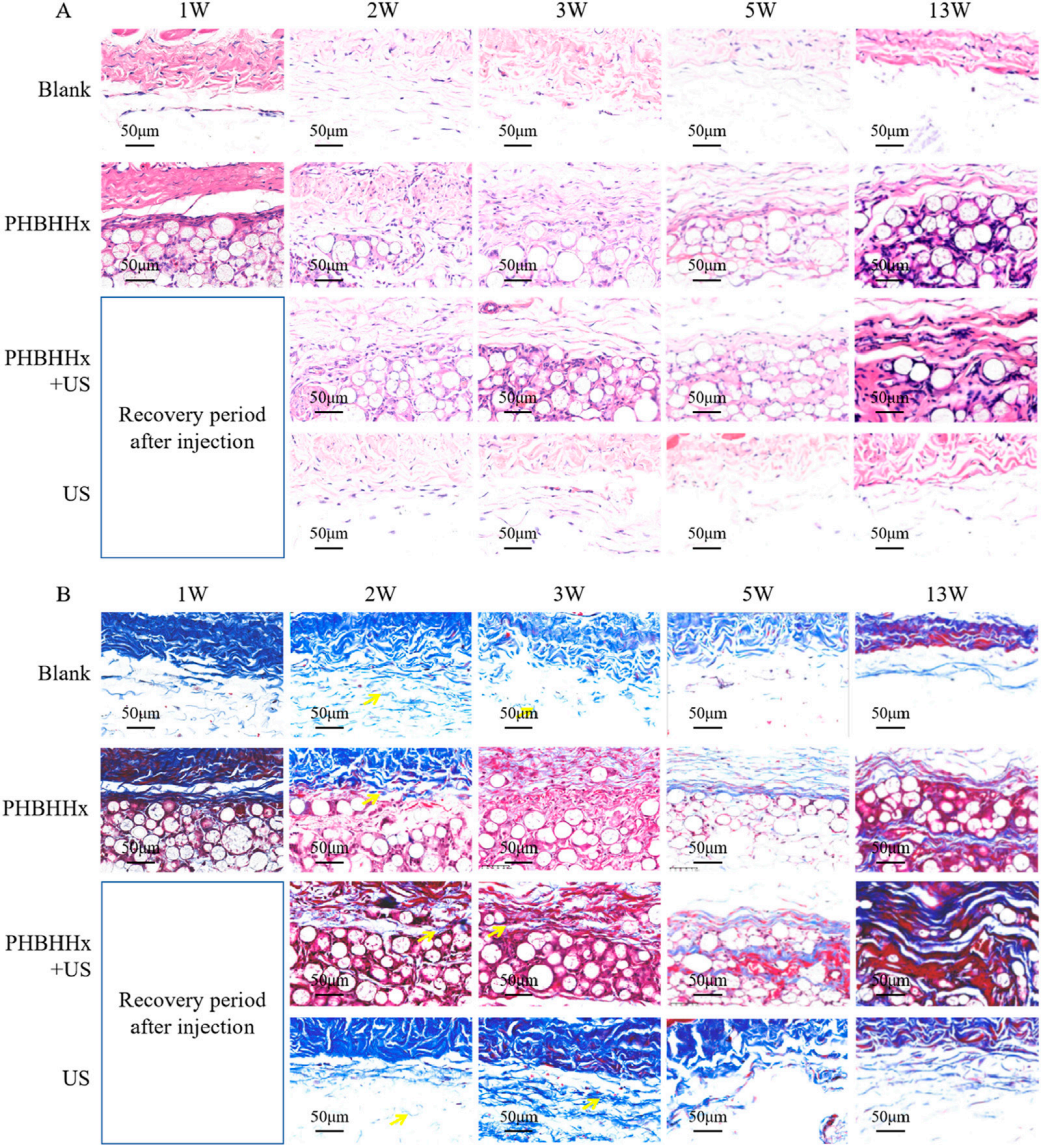
H&E staining **(A)** and Masson’s trichrome staining **(B)** of skin tissue of blank, PHBHHx, PHBHHx + US and US groups.

**FIGURE 6:**
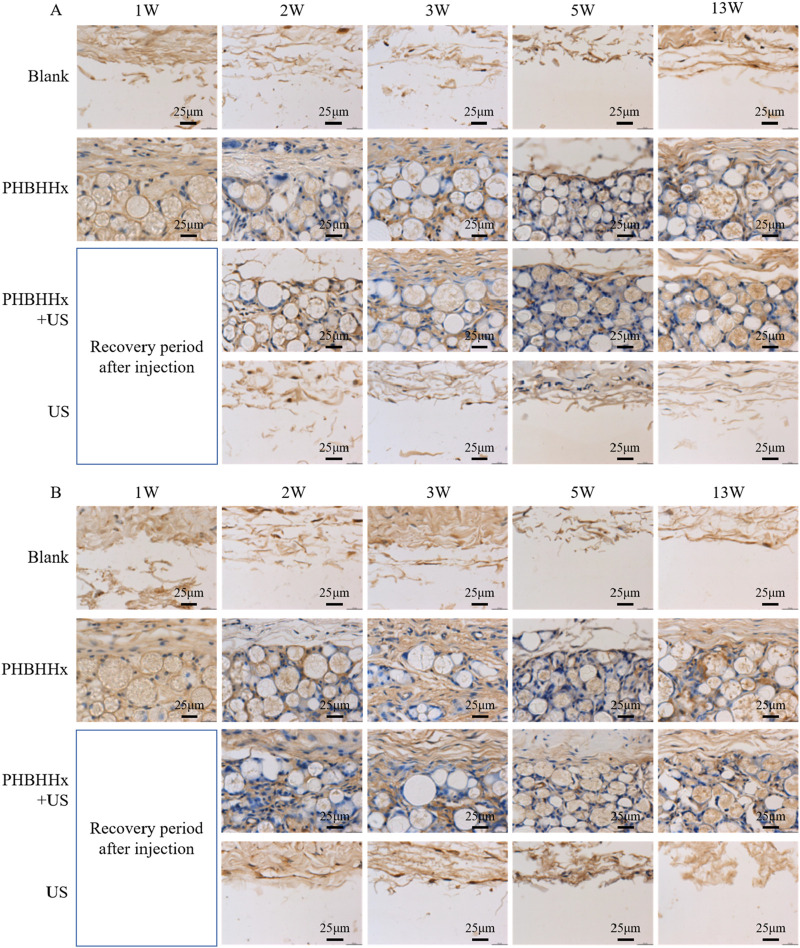
Immunohistochemical staining for Col-I **(A)** and Col-III **(B)** in skin tissues from the blank, PHBHHx, PHBHHx + US and US groups.

**FIGURE 7:**
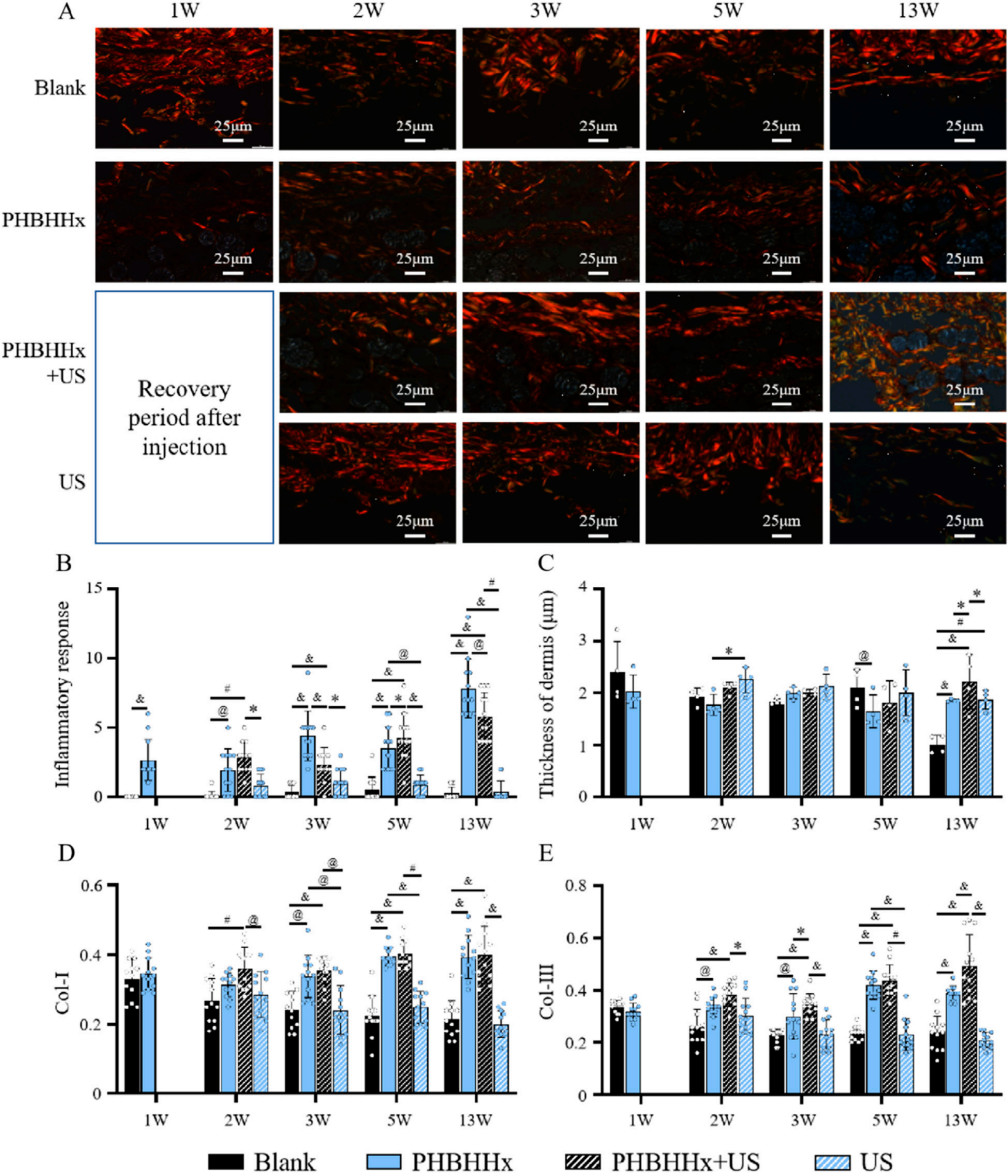
**(A)** Sirus red staining of skin tissue of blank, PHBHHx, PHBHHx + US and US groups. Quantitative evaluation of inflammatory response **(B)** thickness of dermal **(C)** and immunohistochemical [Col-I **(D)** and Col-III **(E)**] analysis of skin tissues from blank, PHBHHx, PHBHHx + US and US groups. The significance of the data was calculated by the one-way ANOVA (*p < 0.05, ^@^p < 0.01, ^#^p < 0.001 and ^&^p < 0.0001).

To evaluate the potential of PHBHHx-CMC composite in promoting collagen regeneration, *in vivo* animal studies were conducted. Histopathological analysis of subcutaneous tissue samples collected at 1, 2, 3, 5, and 13 weeks post-injection revealed distinct differences in tissue response and collagen regeneration across the blank, PHBHHx, PHBHHx + US, and US groups (Figures 5–7). During the early stage (1–5 weeks), all groups exhibited a mild inflammatory response. Compared to the blank group and the US group, the inflammatory response was significantly higher in the PHBHHx and PHBHHx + US groups. However, the overall inflammatory level of PHBHHx and PHBHHx + US groups remained mild. These findings suggested that the PHBHHx-CMC composite demonstrates good biocompatibility with minimal tissue irritation and adverse reactions. By 13 weeks (Figure 5A), the blank group showed no pathological changes, inflammatory infiltration, or granulation tissue formation. The inflammatory response subsided as the saline solution was absorbed into the tissue, restoring it to its normal state. Similarly, no significant pathological changes or notable inflammatory responses were observed in the US group. In contrast, the PHBHHx and PHBHHx + US groups exhibited prominent fibrocystic structures surrounded by significant inflammatory cell infiltration, predominantly lymphocytes (Figure 5A). The heightened inflammatory response in the PHBHHx and PHBHHx + US groups may be attributed to the gradual absorption of CMC by the tissue, facilitating cellular infiltration into the microspheres and enhancing interactions between cells and the biomaterial. Additionally, the formation of fibrotic cystic structures around the implant material likely contributed to the recruitment of immune cells, thereby sustaining the inflammatory response. Compared to the PHBHHx group, the PHBHHx + US group exhibited a significantly reduced inflammatory response (Figure 7B), which may be attributed to the anti-inflammatory effect of LIPUS (Xu et al., 2021).

Masson’s trichrome staining results showed that the collagen staining area in the blank group remained relatively low, indicating a mild tissue response. The collagen staining area in the US group was slightly larger compared to the blank group, which is related to the ability of LIPUS to promote the proliferation of subcutaneous fibroblasts and enhance collagen expression (Figure 5B). Over time, cells gradually infiltrated and penetrated into the material, resulting in a gradual increase in the collagen staining area in the PHBHHx and PHBHHx + US groups. Notably, at weeks 5 and 13, the PHBHHx + US group exhibited significantly deeper and larger collagen staining. In addition, the dermal thickness of the PHBHHx + US group also increased significantly by 13W (Figure 7C). These results indicated that the PHBHHx microspheres, which are the active component within the PHBHHx-CMC composite, significantly enhanced collagen synthesis and regeneration under the stimulation of LIPUS.

Immunohistochemical staining for type I and III collagen further supported these observations, compared to the blank and US groups, the PHBHHx and PHBHHx + US groups exhibited more intense brown staining in the subcutaneous tissue, indicating a more significant collagen synthesis in these groups (Figures 6A, B). Quantitative analysis of immunohistochemical staining revealed that the amount of newly synthesized collagen was significantly higher in the PHBHHx and PHBHHx + US groups compared to the blank and US groups. Moreover, the PHBHHx + US group exhibited a significantly greater collagen content than the PHBHHx group (p < 0.05) (Figures 7D, E). Col-I, which is crucial for tissue strength and structural integrity, and Col-III, involved in early wound healing and tissue remodeling (Singh et al., 2023; Gao et al., 2023), were both substantially elevated in the PHBHHx + US group. These results indicated that LIPUS not only effectively alleviates the inflammatory response but also significantly promotes collagen synthesis induced by PHBHHx microspheres, thereby achieving better filling effects. Furthermore, Sirius Red staining confirmed these findings (Figure 7A),more Col-I and Col-III were found in PHBHHx + US group. Over time, PHBHHx and PHBHHx + US microspheres exhibited prominent staining for type I (strong orange-yellow or bright red) and type III (green) collagen, with a marked increase in the staining of both collagen types in the PHBHHx + US group at week 13.

**FIGURE 8:**
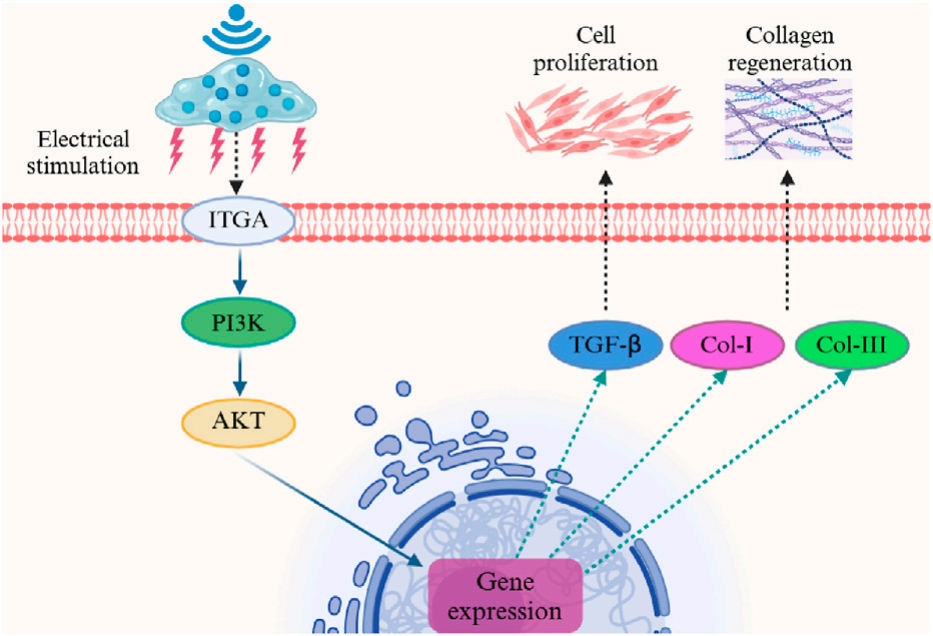
Potential mechanism of PHBHHx -CMC composite for promoting collagen regeneration.

Overall, these results demonstrated that PHBHHx microspheres can effectively stimulated collagen formation, their ability to promote collagen regeneration can be significantly enhanced when stimulated by LIPUS. This suggested that the electrical signals generated by PHBHHx can further regulate cell activity to promote collagen production. Studies have shown that piezoelectric stimulation can promote fibroblast migration, proliferation, and collagen expression by modulating the PI3K/AKT serine/threonine kinase (AKT) pathway (Xu Q. et al., 2024; Dai et al., 2024). The mechanism by which PHBHHx enhances collagen regeneration under ultrasound stimulation may be that the electrical signal generated by PHBHHx under ultrasound stimulation is transmitted from the extracellular matrix (ECM) to intracellular components through the integrin-PI3K pathway. This process activates the PI3K/AKT pathway, which in turn affects the gene expression of downstream molecules such as Col-I, Col-III and TGF-β, thereby promoting cell proliferation and collagen regeneration (Figure 8). These findings highlight the potential of the piezoelectric PHBHHx to improve long-term outcomes in aesthetic applications.

## 4 Conclusion

This study demonstrated the potential of piezoelectric PHBHHx microspheres as a novel biomaterial for enhancing collagen regeneration under LIPUS stimulation. The PHBHHx microspheres, activated by mechanical deformation through external LIPUS, generated localized electrical signals that significantly enhance collagen production. Biocompatibility assessments confirmed that PHBHHx microspheres exhibited no significant cytotoxicity or systemic toxicity, both *in vitro* and *in vivo*. Histological analysis revealed that LIPUS-stimulated PHBHHx group exhibited notably increased collagen deposition, with more organized and denser collagen structures compared to other experimental groups. These findings suggest that the combination of PHBHHx microspheres and LIPUS offers a promising dual-functional strategy, not only enabling volumetric restoration but also actively promoting collagen regeneration through bioelectric cues, which may be linked to the PI3K/AKT pathway. In contrast to conventional dermal fillers that primarily offer temporary volumization, this method promotes endogenous tissue repair and remodeling at a deeper level. Additionally, the inflammatory response in the PHBHHx + US group was well-regulated, further supporting the safety and biocompatibility of the material. The combination of PHBHHx microspheres and LIPUS not only augments collagen production but also mitigates the risk of chronic inflammation, offering a comprehensive solution for soft tissue augmentation and regeneration.

Future research should focus on the scalability of this approach in clinical applications and examine the long-term effects of repeated LIPUS treatments on collagen production and tissue function. Also, the sample size of the animals used in this study is relatively small (n = 2). Hence, increasing the sample size is necessary in the next phase of this project. Such studies will help to further optimize therapeutic efficacy and advance the use of PHBHHx microspheres in aesthetic medicine and regenerative therapies. Overall, the combination of piezoelectric PHBHHx microspheres and LIPUS offers a versatile biomaterial platform with significant potential in soft tissue repair, aesthetic enhancement, and broader regenerative medicine applications. Furthermore, the translational pathway of PHBHHx microspheres from laboratory research to clinical application involves overcoming key challenges such as large-scale production, quality control, and individual variability. Ensuring scalable, cost-effective production while maintaining consistency and adherence to Good Manufacturing Practices (GMP). Rigorous quality control measures, including testing for size uniformity and purity, are essential for reproducibility. Additionally, factors like age, skin type, and immune response can influence treatment outcomes, highlighting the need for personalized treatment plans. Addressing these challenges will be crucial for the successful clinical application of PHBHHx microspheres.

## Data Availability

The original contributions presented in the study are included in the article/Supplementary Material, further inquiries can be directed to the corresponding authors.

## References

[B1] Alghamdi H. Nair S. A. O. Neithalath N. (2019). Insights into material design, extrusion rheology, and properties of 3D-printable alkali-activated fly ash-based binders. Mat. Des. 167, 107634. 10.1016/j.matdes.2019.107634

[B2] Badali V. Checa S. Zehn M. M. Marinkovic D. Mohammadkhah M. (2024). Computational design and evaluation of the mechanical and electrical behavior of a piezoelectric scaffold: a preclinical study. Front. Bioeng. Biotech. 11, 1261108. 10.3389/fbioe.2023.1261108 PMC10808828 38274011

[B3] Bakrani Balani S. Mokhtarian H. Salmi T. Coatanéa E. (2023). An investigation of the influence of viscosity and printing parameters on the extrudate geometry in the material extrusion process. Polymers 15, 2202. 10.3390/polym15092202 37177349 PMC10181141

[B4] Bugnicourt E. Cinelli P. Lazzeri A. Alvarez V. A. (2014). Polyhydroxyalkanoate (PHA): review of synthesis, characteristics, processing and potential applications in packaging, Express. Polym. Lett. 11, 791–808. 10.3144/expresspolymlett.2014.82

[B5] Burgess C. Dayan S. Bank D. Weinkle S. Sartor M. Chawla S. (2024). Hyaluronic acid filler VYC-25L for jawline restoration yields high satisfaction, improved jawline measurements, and sustained effectiveness across skin types, age, and gender for up to 12 Months. Aesthet. Surg. J. sjae172 45, 98–107. 10.1093/asj/sjae172 PMC11634383 39141784

[B6] Dai J. Shao J. Zhang Y. Hang R. Yao X. Bai L. (2024). Piezoelectric dressings for advanced wound healing. J. Mat. Chem. B 12, 1973–1990. 10.1039/D3TB02492J 38305583

[B7] David M. R. Jennifer L. (2021). Skin collagen through the lifestages: importance for skin health and beauty. Plast. Aesthet. Res. 8, 2. 10.20517/2347-9264.2020.153

[B8] Donate R. Paz R. Moriche R. Sayagués M. J. Alemán-Domínguez M. E. Monzón M. (2023). An overview of polymeric composite scaffolds with piezoelectric properties for improved bone regeneration. Mat. Des. 231, 112085. 10.1016/j.matdes.2023.112085

[B9] Fanian F. Deutsch J.-J. Bousquet M. T. Boisnic S. Andre P. Catoni I. (2023). A hyaluronic acid-based micro-filler improves superficial wrinkles and skin quality: a randomized prospective controlled multicenter study. J. Dermatol. Treat. 34, 2216323. 10.1080/09546634.2023.2216323 37577796

[B10] Gao J. Guo Z. Zhang Y. Liu Y. Xing F. Wang J. (2023). Age-related changes in the ratio of Type I/III collagen and fibril diameter in mouse skin. Regen. Biomater. 10, rbac110. 10.1093/rb/rbac110 36683742 PMC9847517

[B11] Huang J. Heng S. Zhang W. Liu Y. Xia T. Ji C. (2022). Dermal extracellular matrix molecules in skin development, homeostasis, wound regeneration and diseases. Semin. Cell. Dev. Biol. 128, 137–144. 10.1016/j.semcdb.2022.02.027 35339360

[B12] Kim J.-E. Sykes J. M. (2011). Hyaluronic acid fillers: history and overview. Facial. Plast. Surg. 27, 523–528. 10.1055/s-0031-1298785 22205525

[B13] Kim J. S. (2021). Fine wrinkle treatment and hydration on the facial dermis using hydrotoxin mixture of microbotox and microhyaluronic acid. Aesthet. Surg. J. 4, 538–549. 10.1093/asj/sjaa231 PMC8240748 32779694

[B14] Li A. R. Taylor B. Snyder A. N. Schlesinger T. (2023). Algeness—what do we know about this filler. Dermatol. Revs. 4, 100–104. 10.1002/der2.184

[B15] Li Y. Fan Y. Zhao S. Cheng B. (2024). Ultrasound-triggered piezoelectric polyetheretherketone with boosted osteogenesis via regulating Akt/GSK3β/β-catenin pathway. J. Nanobiotechnol. 22, 539. 10.1186/s12951-024-02814-9 PMC11375992 39237993

[B16] Lizarraga Valderrama L. R. Thomas C. Cadiz Miranda J. I. Roy I. (2018). Tissue engineering: polyhydroxyalkanoate-based materials and composites, encyclopedia of polymer applications. Boca Raton, FL, USA: Taylor and Francis Group, 2652–2675.

[B17] Luo W. Li Z. Ren C. Xu H. Zhang H. Cao Z. (2024). Zn^2+^ driven H2S/Cu^2+^ sustained releasing nanofibers with immunoregulation for wound healing. Mat. Des. 238, 112626. 10.1016/j.matdes.2023.112626

[B18] Miyazaki T. Honda A. Ikegami T. Iwamoto J. Monma T. Hirayama T. (2015). Simultaneous quantification of salivary 3-hydroxybutyrate, 3-hydroxyisobutyrate, 3-hydroxy-3-methylbutyrate, and 2-hydroxybutyrate as possible markers of amino acid and fatty acid catabolic pathways by LC-ESI-MS/MS. SpringerPlus 4, 494. 10.1186/s40064-015-1304-0 26389019 PMC4571036

[B19] Narins R. S. Dayan S. H. Brandt F. S. Baldwin E. K. (2008). Persistence and improvement of nasolabial fold correction with nonanimal‐stabilized hyaluronic acid 100,000 gel particles/mL filler on two retreatment schedules: results up to 18 months on two retreatment schedules. Dermatol. Surg. 34, S2–S8. 10.1111/j.1524-4725.2008.34236.x 18547177

[B20] Panda A. K. Basu B. (2021). Biomaterials-based bioengineering strategies for bioelectronic medicine. Mat. Sci. Eng. R. 146, 100630. 10.1016/j.mser.2021.100630

[B21] Pierre S. Liew S. Bernardin A. (2015). Basics of dermal filler rheology. Dermatol. Surg. 41, S120–S126. 10.1097/DSS.0000000000000334 25828036

[B22] Pouton C. W. Akhtar S. (1996). Biosynthetic polyhydroxyalkanoates and their potential in drug delivery. Rev 18, 133–162. 10.1016/0169-409X(95)00092-L

[B23] Pu S.-Y. Huang Y.-L. Pu C.-M. Kang Y.-N. Hoang K. D. Chen K.-H. (2080). Effects of oral collagen for skin anti-aging: a systematic review and meta-analysis. Nutrients 15, 2080. 10.3390/nu15092080 PMC10180699 37432180

[B24] Rajabi A. H. Jaffe M. Arinzeh T. L. (2015). Piezoelectric materials for tissue regeneration: a review. Biomater 24, 12–23. 10.1016/j.actbio.2015.07.010 26162587

[B25] Ranella A. Barberoglou M. Bakogianni S. Fotakis C. Stratakis E. (2010). Tuning cell adhesion by controlling the roughness and wettability of 3D micro/nano silicon structures. Biomater 6, 2711–2720. 10.1016/j.actbio.2010.01.016 20080216

[B26] Shin S. H. Lee Y. H. Rho N.-K. Park K. Y. (2023). Skin aging from mechanisms to interventions: focusing on dermal aging. Front. Physiol. 14, 1195272. 10.3389/fphys.2023.1195272 37234413 PMC10206231

[B27] Singh D. Rai V. Agrawal D. K. (2023). Regulation of collagen I and collagen III in tissue injury and regeneration. Cardiovasc. Med. 7, 5–16. 10.26502/fccm.92920302 PMC9912297 36776717

[B28] Tang X. Duan Q. Chen Y. Yi Z. Jiang H. Ni Y. (2024). Encapsulated inorganic pigments in epoxy composite microspheres using emulsion synthesis. Colloid. Surf. A 701, 134963. 10.1016/j.colsurfa.2024.134963

[B29] Wang Q. Yan H. Yao L. Li W. Xiao J. (2024). A highly durable and biocompatible bionic collagen implant with exceptional anti-calcification and collagen regeneration capabilities for improved skin rejuvenation. Mat. Des. 244, 113177. 10.1016/j.matdes.2024.113177 38629894

[B30] Xu H. Zhang Y. Zhang Y. Zhao Z. Xue T. Wang J. (2024a). 3D bioprinting advanced biomaterials for craniofacial and dental tissue engineering – a review. Mat. Des. 241, 112886. 10.1016/j.matdes.2024.112886

[B31] Xu M. Wang L. Wu S. Dong Y. Chen X. Wang S. (2021). Review on experimental study and clinical application of low-intensity pulsed ultrasound in inflammation. Imaging. Med. Surg. 11, 443–462. 10.21037/qims-20-680 PMC7719927 33392043

[B32] Xu Q. Dai W. Li P. Li Q. Gao Z. Wu X. (2024b). Piezoelectric film promotes skin wound healing with enhanced collagen deposition and vessels regeneration via upregulation of PI3K/AKT. Nano. Res. 17, 7461–7478. 10.1007/s12274-024-6717-z

[B33] Yang C.-Y. Chang Y.-C. Tai H.-C. Liao Y.-H. Huang Y.-H. Hui R.C.-Y. (2024). Evaluation of collagen dermal filler with lidocaine for the correction of nasolabial folds: a randomized, double-blind, multicenter clinical trial. Clin. Cosmet. Inv. Derm. 17, 1621–1631. 10.2147/CCID.S447760 PMC11244637 39006129

[B34] Yang L. Zhao Y. Cui D. Liu Y. Zou Q. Xu S. (2022). Coaxial bioelectrospinning of P34HB/PVA microfibers biomimetic scaffolds with simultaneity cell-laden for improving bone regeneration. Mat. Des. 213, 110349. 10.1016/j.matdes.2021.110349

[B35] Zhang C. Song W. Guo X. Li Z. Kong Y. Du J. (2025). Piezoelectric nanocomposite electrospun dressings: tailoring mechanics for scar-free wound recovery. Biomater. Adv. 167, 214119. 10.1016/j.bioadv.2024.214119 39556886

[B36] Zhang Q. An Z.-Y. Jiang W. Jin W.-L. He X.-Y. (2023). Collagen code in tumor microenvironment: functions, molecular mechanisms, and therapeutic implications. Biomed. Pharmacother. 166, 115390. 10.1016/j.biopha.2023.115390 37660648

[B37] Zheng F. Wu T. Wang F. Li H. Tang H. Cui X. (2024). Low-intensity pulsed ultrasound promotes the osteogenesis of mechanical force-treated periodontal ligament cells via Piezo1. Front. Bioeng. Biotech. 12, 1347406. 10.3389/fbioe.2024.1347406 PMC11061374 38694622

