# Supplementary material for: Piezoelectric poly(3-hydroxybutyrate-co-3-hydroxyhexanoate) (PHBHHx) microspheres for collagen regeneration and skin rejuvenation
Source: Front Bioeng Biotechnol. 2025 Mar 12;13:1554825. doi: 10.3389/fbioe.2025.1554825 (PMC11937035; doi:10.3389/fbioe.2025.1554825)
Supplement: Supplementary file 1 [file DataSheet1.docx]

Table S1. Effect on body weight of rats (x̄ ±SD.g)

| Week | Male control group | Male sample group | Female control group | Female sample group |
| --- | --- | --- | --- | --- |
| 0 | 186.00 ± 7.77 | 187.08 ± 7.21 | 165.65 ±6.47 | 166.11 ± 5.84 |
| 1 | 252.58 ± 6.43 | 254.69 ± 8.59 | 201.24 ±11.23 | 202.15 ± 12.49 |
| 2 | 320.85 ± 8.40 | 325.42 ± 9.28 | 227.91 ±12.38 | 233.83 ± 14.56 |
| 3 | 372.76 ± 13.18 | 381.30 ± 11.64 | 243.35 ±16.39 | 248.94 ± 15.00 |
| 4 | 417.62 ± 14.50 | 419.93 ± 12.30 | 264.65 ±18.21 | 264.14 ± 17.87 |
| 5 | 451.81 ± 19.84 | 453.18 ± 15.26 | 267.65 ±17.65 | 270.25 ± 22.31 |
| 6 | 484.65 ± 24.04 | 482.74 ± 18.24 | 280.06 ±16.98 | 276.59 ± 22.34 |
| 7 | 512.25 ± 29.23 | 511.40 ± 20.90 | 286.65 ±16.68 | 283.46 ± 24.52 |
| 8 | 530.52 ± 33.10 | 532.48 ± 22.91 | 289.91 ±16.64 | 288.67 ± 25.41 |
| 9 | 552.26 ± 40.70 | 551.59 ± 27.39 | 295.51 ±18.36 | 297.03 ± 21.84 |
| 10 | 560.00 ± 44.44 | 567.74 ± 27.86 | 296.58 ±18.36 | 298.53 ± 21.35 |
| 11 | 580.65 ± 42.54 | 582.47 ± 27.76 | 304.30 ±18.81 | 303.33 ± 19.80 |
| 12 | 592.91 ± 44.30 | 593.23 ± 32.88 | 310.60 ±20.80 | 309.50 ± 21.05 |
| 13 | 600.06 ± 43.45 | 592.38 ± 21.91 | 313.57 ±20.54 | 311.86 ± 21.92 |

Note: Compared with the control group, P>0.05


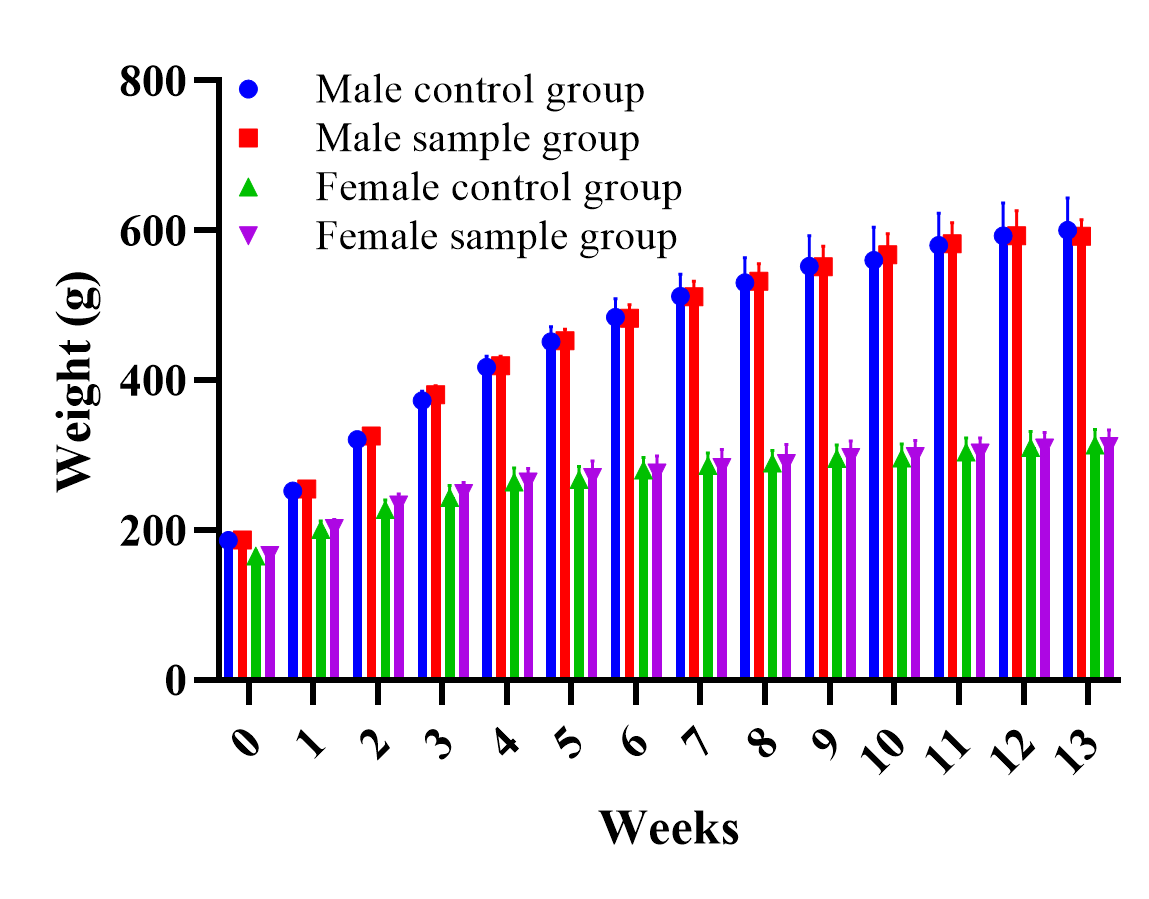


Figure S1. Effect on body weight of rats

Table S2. Effects on feed consumption in rats (x̄ ±SD.g/100g·24h)

| Week | Male control group | Male sample group | Female control group | Female sample group |
| --- | --- | --- | --- | --- |
| 0 | 13.02 ± 0.94 | 12.97 ± 1.05 | 11.23 ±0.88 | 11.73 ± 0.92 |
| 1 | 12.47 ± 0.92 | 11.77 ± 0.80 | 10.31 ±0.36 | 10.61 ± 0.30 |
| 2 | 8.09 ± 0.29 | 8.42 ± 0.34 | 8.83 ±0.50 | 8.71 ± 0.14 |
| 3 | 8.09 ± 0.29 | 8.42 ± 0.34 | 8.83 ±0.50 | 8.71 ± 0.14 |
| 4 | 7.74 ± 0.48 | 7.62 ± 0.19 | 8.72 ±0.28 | 10.47 ± 3.93 |
| 5 | 6.28 ± 0.48 | 5.86 ± 0.37 | 5.50 ±0.27 | 6.02 ± 0.58 |
| 6 | 6.65 ± 0.43 | 6.66 ± 0.28 | 6.48 ±0.24 | 6.54 ± 0.54 |
| 7 | 6.18 ± 0.37 | 5.78 ± 0.24 | 6.21 ±0.50 | 6.49 ± 0.55 |
| 8 | 5.70 ± 0.20 | 5.56 ± 0.23 | 5.95 ±0.35 | 6.33 ± 0.31 |
| 9 | 5.59 ± 0.24 | 5.35 ± 0.11 | 5.92 ±0.52 | 6.51 ± 0.48 |
| 10 | 5.25 ± 0.16 | 5.09 ± 0.21 | 5.75 ±0.20 | 5.75 ±0.20 |
| 11 | 5.15 ± 0.36 | 4.94 ± 0.28 | 5.54 ±0.41 | 5.79 ± 0.41 |
| 12 | 5.58 ± 0.17 | 5.39 ± 0.27 | 6.06 ±0.34 | 6.49 ± 0.31 |
| 13 | 4.63 ± 0.29 | 4.52 ± 0.15 | 5.24 ±0.61 | 5.31 ± 0.47 |

Note: Compared with the control group, P>0.05


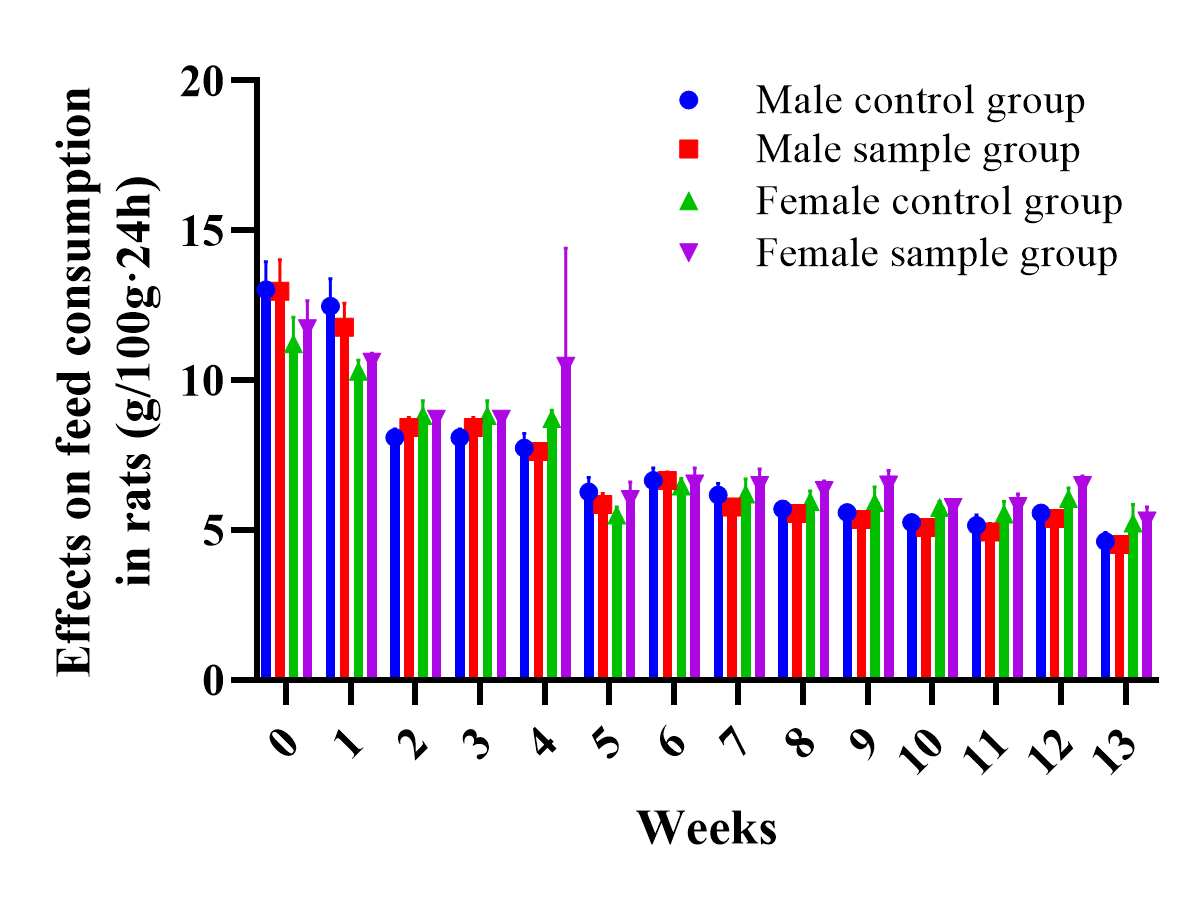


Figure S2. Effects on feed consumption in rats

Table S3. Effects on rat hematology (x̄ ± SD)

| Items | Unit | Male control group | Male sample group | Female control group | Female sample group |
| --- | --- | --- | --- | --- | --- |
| WBC | 10^9^/L | 7.55±1.00 | 5.92 ± 1.02 | 3.74 ±1.28 | 3.04 ± 0.91 |
| RBC | 10^12^/L | 8.75 ± 0.34 | 8.63 ± 0.40 | 8.06 ±0.43 | 8.29 ± 0.42 |
| HGB | g/L | 155.33 ± 4.97 | 155.00 ± 6.78 | 151.80 ±5.81 | 155.90 ± 5.36 |
| HCT | % | 44.17 ± 1.52 | 44.53 ± 2.78 | 43.67 ±1.44 | 44.55 ± 1.82 |
| PLT | 10^9^ /L | 1207.00 ± 68.88 | 1252.10 ± 72.04 | 1078.50 ±91.91 | 1092.70 ± 153.44 |
| MCV | fL | 50.49 ± 1.29 | 51.59 ± 1.98 | 54.22 ±1.52 | 53.78 ± 1.65 |
| MCH | pg | 17.76 ± 0.33 | 17.94 ± 0.45 | 18.85 ±0.55 | 18.81 ± 0.50 |
| MCHC | g/L | 351.67 ± 4.61 | 348.60 ± 8.06 | 347.50 ±5.60 | 349.90 ± 5.53 |
| NEUT | 10^9^ /L | 1.61 ± 0.76 | 1.41 ± 0.54 | 0.60 ±0.23 | 0.52 ± 0.23 |
| LYMPH | 10^9^/L | 5.74 ± 1.23 | 3.36 ± 1.26** | 2.86 ±1.06 | 2.31 ± 0.78 |
| MONO | 10^9^/L | 0.38 ± 0.22 | 0.29 ± 0.14 | 0.18 ±0.11 | 0.13 ± 0.06 |
| EO | 10^9^ /L | 0.15 ± 0.05 | 0.16 ± 0.07 | 0.11 ±0.05 | 0.07 ± 0.03 |
| BASO | 10^9^ /L | 0.00 ± 0.00 | 0.00 ± 0.00 | 0.00 ± 0.00 | 0.00 ± 0.00 |
| NEUT | % | 20.40 ± 7.61 | 27.93 ± 5.59* | 16.71 ±5.84 | 18.03 ± 7.63 |
| LYMPH | % | 72.94 ± 9.48 | 63.47 ± 5.96* | 75.78 ±6.31 | 75.31 ± 8.09 |
| MONO | % | 4.68 ± 1.98 | 5.42 ± 1.34 | 4.60 ±1.87 | 4.11 ± 0.99 |
| EO | % | 1.98 ± 0.70 | 3.18 ± 0.95** | 2.91 ±0.83 | 2.55 ± 1.23 |
| BASO | % | 0.00 ± 0.00 | 0.00 ± 0.00 | 0.00 ± 0.00 | 0.00 ± 0.00 |
| PT | Sec | 10.50 ± 0.26 | 10.43 ± 0.26 | 9.68 ±0.15 | 9.80 ± 0.44 |
| APTT | Sec | 16.63 ± 0.92 | 17.71 ± 1.66 | 15.26 ±1.26 | 17.00 ± 1.59* |

Note: * Compared with the control group of the same gender P<0.05; ** compared with the control group of the same gender P<0.01;

Reference intervals: male LYMPH10^9^/L(0.55-9.04), NEUT%(0.20-41.22), LYMPH% (53.68-93.83), EO%(0.00-4.54); APTT Sec (13.71-19.54) in females


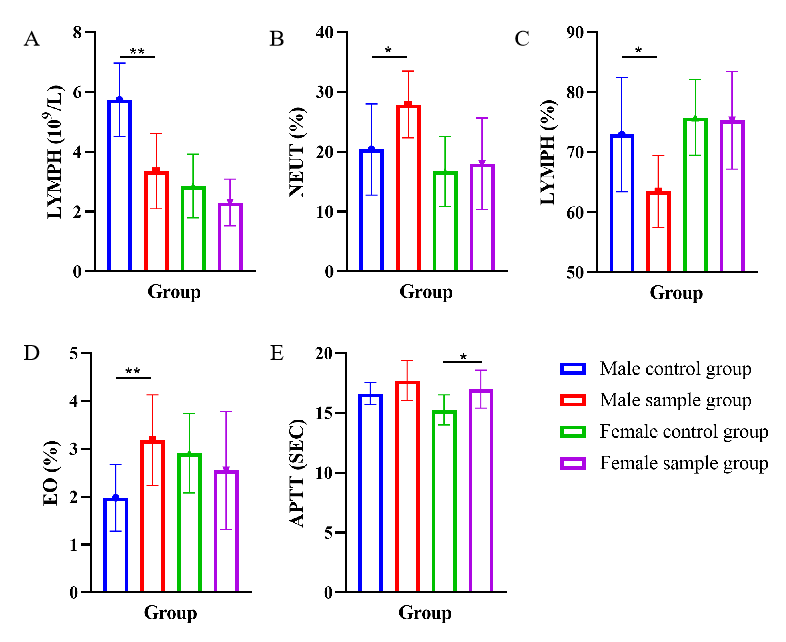


Figure S3. Effects on rat hematology. (A) The absolute value of lymphocytes. The percentages of neutrophil polymorphonuclear cell (B) and lymphocyte (C). The percentage of eosinophils (EO%) (D). The activated partial thromboplastin time (E).

Table S4. Effects on biochemical parameters in rats (x̄ ± SD).

| Items | Unit | Male control group | Male sample group | Female control group | Female sample group |
| --- | --- | --- | --- | --- | --- |
| ALT | U/L | 47.22 ± 30.45 | 40.50 ± 13.36 | 85.30 ± 44.67 | 32.60 ± 11.97** |
| AST | U/L | 218.22 ± 216.23 | 186.30 ± 34.03 | 218.70 ± 68.76 | 176.90 ± 75.88* |
| ALP | U/L | 68.33 ± 12.76 | 63.50 ± 11.40 | 34.70 ± 6.36 | 28.00 ± 6.94 |
| GlU | m mol/L | 8.06 ± 1.72 | 7.71 ± 0.96 | 6.00 ± 1.37 | 5.68 ± 1.58 |
| UREA | m mol/L | 9.24 ± 1.42 | 8.95 ± 1.37 | 9.66 ± 1.33 | 9.41 ± 1.10 |
| TG | m mol/L | 0.49 ± 0.19 | 0.51 ± 0.18 | 0.49 ± 0.11 | 0.53 ± 0.11 |
| CHO | m mol/L | 2.22 ± 0.40 | 2.20 ± 0.38 | 2.44 ± 0.34 | 2.10 ± 0.68 |
| TP | g/L | 59.08 ± 1.81 | 60.02 ± 3.18 | 62.73 ± 2.85 | 62.94 ± 2.79 |
| ALB | g/L | 21.62 ± 0.85 | 22.00 ± 1.58 | 25.47 ± 1.50 | 26.15 ± 1.84 |
| Ca | m mol/L | 2.20 ± 0.13 | 2.32 ± 0.21 | 2.37 ± 0.18 | 2.28 ± 0.13 |
| P | m mol/L | 2.42 ± 0.27 | 2.71 ± 0.48 | 1.89 ± 0.36 | 2.22 ± 0.43 |
| Cl | m mol/L | 101.05 ± 1.62 | 101.22 ± 0.92 | 103.02 ± 0.70 | 102.62 ± 1.44 |
| CRE | μ mol/L | 57.78 ± 10.33 | 62.60 ± 8.96 | 66.30 ± 10.33 | 61.90 ± 9.61 |
| K | m mol/L | 5.84 ± 0.46 | 6.34 ± 0.90 | 5.25 ± 0.32 | 5.32 ± 0.39 |
| Na | m mol/L | 145.44 ± 1.93 | 145.88 ± 0.91 | 146.43 ± 1.35 | 146.94 ± 1.00 |

Note: * Compared with the control group of the same gender P<0.05; ** compared with control group of the same sex P<0.01.

Reference intervals: ALT (17.11-252.80), AST (52.74-777.74) in females.


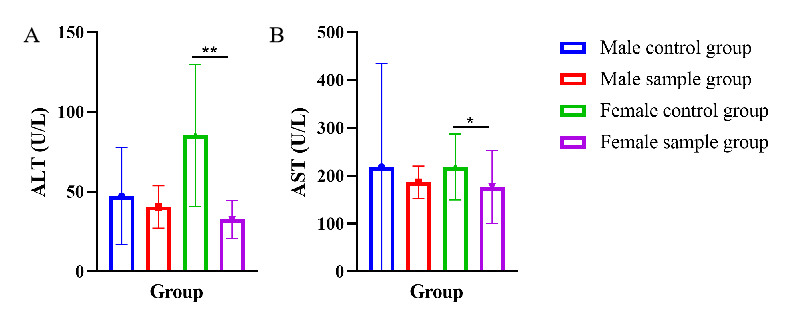


Figure S4. Effects on biochemical parameters in rats. (A) Alanine aminotransferase. (B) Aspartate aminotransferase

Table S5. Effects on wet weight of organs in rats (x̄ ± SD.g)

| Internal organs | Male control group | Male sample group | Female control group | Female sample group |
| --- | --- | --- | --- | --- |
| Brain | 1.99 ±0.11 | 2.00 ± 0.14 | 1.92 ± 0.09 | 1.84 ± 0.13 |
| Heart | 1.53 ±0.17 | 1.51 ± 0.16 | 0.86 ± 0.07 | 0.85 ± 0.06 |
| Liver | 15.52 ±3.63 | 16.01 ± 1.53 | 7.16 ± 0.95 | 7.23 ± 0.75 |
| Spleen | 0.91 ±0.12 | 0.96 ± 0.13 | 0.52 ± 0.11 | 0.50 ± 0.09 |
| Renal | 3.92 ±0.57 | 3.95 ± 0.45 | 2.00 ± 0.13 | 2.08 ± 0.16 |
| Adrenal glands | 0.06 ±0.02 | 0.07 ± 0.01 | 0.07 ± 0.01 | 0.07 ± 0.02 |
| Testes/Uterus | 3.52 ±0.31 | 3.28 ± 0.38 | 0.77 ± 0.27 | 0.72 ± 0.21 |
| Epididymis/ovary | 1.65 ±0.24 | 1.47 ± 0.16 | 0.16 ± 0.05 | 0.15 ± 0.03 |
| Thymus gland | 0.52 ±0.11 | 0.53 ± 0.12 | 0.40 ± 0.12 | 0.43 ± 0.10 |

Note: Compared with the control group, P>0.05.

Table S6. Effects on organ coefficients in rats (x̄ ± SD.g/100g)

| Internal organs | Male control group | Male sample group | Female control group | Female sample group |
| --- | --- | --- | --- | --- |
| Brain | 0.35 ±0.02 | 0.35 ±0.03 | 0.67 ± 0.05 | 0.65 ± 0.07 |
| Heart | 0.27 ±0.03 | 0.26 ±0.03 | 0.30 ± 0.02 | 0.30 ± 0.03 |
| Liver | 0.30 ± 0.03 | 2.78 ±0.30 | 2.51 ± 0.26 | 2.55 ± 0.31 |
| Spleen | 0.16 ±0.02 | 0.17 ±0.03 | 0.18 ± 0.04 | 0.18 ± 0.03 |
| Renal | 0.68 ±0.07 | 0.69 ±0.09 | 0.70 ± 0.03 | 0.73 ± 0.06 |
| Adrenal glands | 0.0052 ±0.0011 | 0.0058 ±0.0013 | 0.0114 ± 0.0020 | 0.0118 ± 0.0036 |
| Testes/Uterus | 0.61 ±0.06 | 0.57 ±0.08 | 0.27 ± 0.09 | 0.25 ± 0.06 |
| Epididymis/ovary | 0.29 ±0.05 | 0.26 ±0.03 | 0.06 ± 0.02 | 0.05 ± 0.01 |
| Thymus gland | 0.09 ±0.02 | 0.09 ±0.02 | 0.14 ± 0.04 | 0.15 ± 0.03 |

Note: Compared with the control group, P>0.05

Table S7. Summary of pathological examination results of subchronic toxicity test in rats

|  |  | Sum | 120 | | 0/20 | |
| --- | --- | --- | --- | --- | --- | --- |
| Lung | Foam cell foci | + | 1/10 | 0/10 | 0/10 | 0/10 |
|  |  | Sum | 1/20 | | 0/20 | |
| Renal | Interstitial inflammation | + | 0/10 | 1/10 | 0/10 | 0/10 |
|  |  | Sum | 1/20 | | 0/20 | |
| Testicles | Some spermatogenic cells disappeared | ++ | 0/10 | 0/10 | 0/10 | 1/10 |
|  |  | Sum | 0/20 | | 1/20 | |
| Perineum | Epidermoid cyst | + | 0/10 | 0/10 | 1/10 | 0/10 |
|  |  | Sum | 0/20 | | 1/20 | |
| Urinary bladder | Erosion of mucosa | ++ | 0/10 | 0/10 | 0/10 | 2/10 |
|  |  | Sum | 0/20 | | 2/20 | |
| eyeball | Deformation of crystal | ++ | 0/10 | 0/10 | 0/10 | 1/10 |
|  |  | Sum | 0/20 | | 1/20 | |
| Subcutaneous implantation site | Foreign body granulomatous inflammation | +++ | 0/10 | 0/10 | 10/10 | 10/10 |
|  |  | Sum | 0/20 | | 20/20 | |

+ slight reaction; ++mild reaction; +++moderate reaction; ++++severe reaction
